# Sifting through genomes with iterative-sequence clustering produces a large, phylogenetically diverse protein-family resource

**DOI:** 10.1186/1471-2105-13-264

**Published:** 2012-10-13

**Authors:** Thomas J Sharpton, Guillaume Jospin, Dongying Wu, Morgan GI Langille, Katherine S Pollard, Jonathan A Eisen

**Affiliations:** 1 The J. David Gladstone Institutes, University of California San Francisco, San Francisco, CA, 94158, USA; 2 UC Davis Genome Center, University of California, Davis, Davis, CA, 95616, USA; 3 Department of Biochemistry & Molecular Biology, Dalhousie University, Halifax, Nova Scotia, Canada; 4 Department of Epidemiology & Biostatistics, Institute for Human Genetics, University of California San Francisco, San Francisco, CA, 94158, USA; 5 Deptartment of Evolution and Ecology, University of California, Davis, Davis, CA, 95616, USA; 6 Deptartment of Medical Microbiology and Immunology, University of California, Davis, Davis, CA, 95616, USA; 7 Department of Energy Joint Genome Institute, Walnut Creek, CA, 94598, USA

## Abstract

**Background:**

New computational resources are needed to manage the increasing volume of biological data from genome sequencing projects. One fundamental challenge is the ability to maintain a complete and current catalog of protein diversity. We developed a new approach for the identification of protein families that focuses on the rapid discovery of homologous protein sequences.

**Results:**

We implemented fully automated and high-throughput procedures to *de novo* cluster proteins into families based upon global alignment similarity. Our approach employs an iterative clustering strategy in which homologs of known families are sifted out of the search for new families. The resulting reduction in computational complexity enables us to rapidly identify novel protein families found in new genomes and to perform efficient, automated updates that keep pace with genome sequencing. We refer to protein families identified through this approach as “Sifting Families,” or SFams. Our analysis of ~10.5 million protein sequences from 2,928 genomes identified 436,360 SFams, many of which are not represented in other protein family databases. We validated the quality of SFam clustering through statistical as well as network topology–based analyses.

**Conclusions:**

We describe the rapid identification of SFams and demonstrate how they can be used to annotate genomes and metagenomes. The SFam database catalogs protein-family quality metrics, multiple sequence alignments, hidden Markov models, and phylogenetic trees. Our source code and database are publicly available and will be subject to frequent updates (http://edhar.genomecenter.ucdavis.edu/sifting_families/).

## Background

Advances in DNA sequencing technology have expedited the rate at which new genomes are being sequenced. While this is a tremendous benefit for biology, this deluge of genome sequences presents new challenges for the management, distribution and analysis of data. New computational approaches are required to efficiently analyze biological data in a timely fashion.

Genome sequences form the basis for our significantly improved understanding of the diversity of proteins. As genomes are sequenced, they reveal both new versions of known proteins, helping us to construct “families” of related but distinct proteins, as well as representatives of previously unknown protein families [1]. This is especially the case for microorganisms, as even very closely related taxa can have significant differences in protein families [2-4]. Recent genomic [5] and metagenomic [6] analyses suggest that we have yet to discover a tremendous amount of the protein diversity that exists in nature.

Protein family databases serve to define our knowledge of protein diversity. Many protein family databases have been developed, including COGs [7], UniProtKB/Swiss-Prot [8], the KEGG [9], HAMAP [10], FIGfams [11], PFAM [12], TIGRFAM [13], Panther [14], PhyloFacts [15], ProtClustDB [16] and EggNOG [17]. Protein databases differ in a variety of ways, including how protein families are defined, the phylogenetic diversity represented in the database, the method by which protein families are identified, and the format in which family data is encoded. These resources have proven to be critical components of genome and metagenome annotation [6,18], evolutionary analysis (e.g., the evolution of biological functions [19]), and community ecology (e.g., the functional assortment of communities [20]). Maintaining an up-to-date catalog of protein family diversity is important to the development of these research fields.

While these databases have been and remain useful resources for biological sequence analysis, they are generally not designed to be able to rapidly discover and accommodate new protein families that may be revealed through the sequencing of new genomes. For example, most databases invoke some amount of curation in the process of identifying families. Curation improves the precision of data, but results in a bottleneck when processing new sequences. In addition, when updating their catalog of protein family diversity, many databases *de novo* cluster all sequences, including those that were previously identified as being members of a family. While this helps ensure optimal clustering of sequences into families, it can present substantial computational challenges in the face of ever-increasing amounts of data.

Given the infrastructural challenges that result from increased rates of sequencing, we reasoned that an alternative protein family identification strategy might be warranted. With the goal of maximizing the coverage of protein family diversity, we developed a high-throughput procedure for identifying protein families that focuses on efficiency, automation, and updatability. Specifically, we employ a fully automated method to cluster sequences into families using as much starting data as possible. This has the benefits of minimizing the curation bottleneck and producing families that are not biased towards historical artifacts, such as human-created labels that interfere with what the data supports. In-depth curation is left to researchers if a particular family is deemed relevant in a scientific analysis. Additionally, we increase the ease of updating the database by decreasing the computational complexity associated with processing new data. This is accomplished by an iterative clustering procedure that uses previously identified families to sift out homologs of known families in new data. Only the remaining sequences are then subject to *de novo* clustering to identify new families. This process gives rise to what we call “Sifting Families”, or SFams for short.

Here, we outline our strategy for creating SFams in an automated manner given any set of protein sequences. We also describe a computational pipeline that executes this strategy and produces a database of 436,360 SFams based upon sequences from 2,928 genome sequences from all three domains of life (over 95% of which are bacterial). We demonstrate how SFams can be rapidly updated given new genomic data. We use SFams to annotate genomes and metagenomes by classifying sequences into SFams. We find that SFams cover most of the diversity in new genomes and recover a greater number of metagenomic homologs than alternative HMM-based protein family databases. Our SFam identification software and database is publically available for download (http://edhar.genomecenter.ucdavis.edu/sifting_families/).

## Results and discussion

### An iterative approach to defining protein families

**Figure 1:**
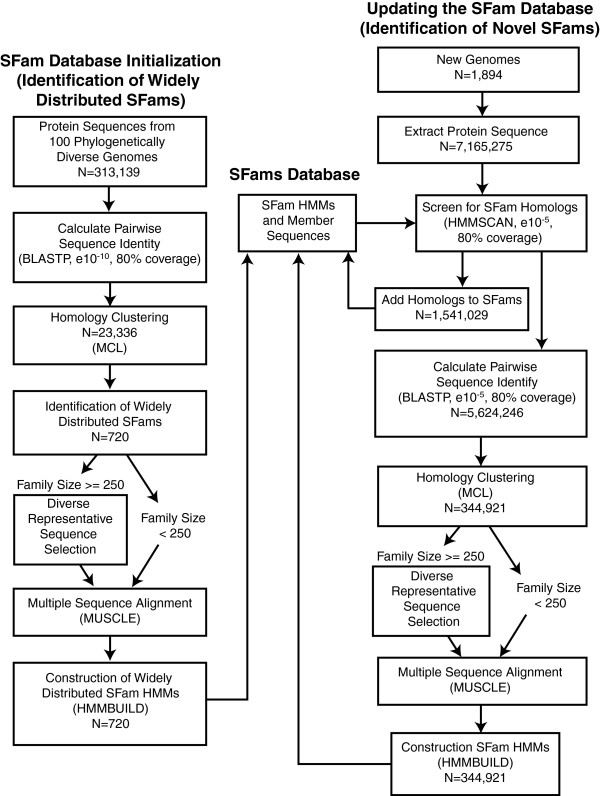
**A Computation Workflow to Rapidly Identify and Update SFams. **This workflow illustrates the general steps (boxes) used to initialize (left) and update (right) the database of SFams (center). Where appropriate, the algorithms used at each step are listed in parenthetical statements as are the e-value (*e.g.*, e10^-10^) and coverage thresholds (*e.g.*, 80%) used to infer homology between a pair of sequences or a sequence and an HMM. The number of sequences or HMMs considered at various steps is also listed (*e.g.*, N=720). The SFam database was initialized by identifying 720 *de novo* clustered families that are found in 50% of the 100 phylogenetically diverse representative Bacterial and Archaeal genomes that we selected. The similarity between all pairs of protein sequences from these genomes was calculated and used to cluster proteins into families. Each SFam’s sequences were then aligned and used to train Hidden Markov Models (HMMs). These HMMs were then used to screen for homologs among the ~7 million protein sequences found in the 1,894 genomes we originally downloaded, which include the 100 representative families. Detected homologs were added to the database of previously identified SFams. All protein sequences in the database that are not SFam members were then subject to independent *de novo* clustering and HMM construction. A similar iterative approach is used to annotate new genome sequences.

We developed an easily implemented solution that generates families from protein sequences that exhibit global homology and allows for relatively rapid and regular updates as new genomes are sequenced (Figure 1). Our procedure uses the Markov clustering algorithm (MCL) with pairwise BLASTP percent sequence identity as a distance measure to group protein sequences into families. MCL is a fast and scalable unsupervised *de novo* clustering algorithm [21]. We build a MySQL database that includes an alignment, a profile-Hidden Markov Model (HMM), and a phylogenetic tree to describe the diversity of each family. These resources can be used for a variety of research problems, including annotating protein sequences according to similarity to the HMMs and studying the relationships between protein families.

To update the families with new sequence data (e.g., from recently sequenced genomes), we assign each new protein sequence to an existing family if it has high enough similarity to the family HMM (see Methods). All sequences with no significant match in the current database are clustered with MCL to identify novel families, which we call Sifting Families (i.e., SFams). This process can be periodically repeated. Our iterative strategy limits the number of sequences that need to be clustered at each update, enabling the *de novo* identification of large numbers of diverse protein families in a computationally feasible manner.

### Generating the SFam database

We applied our approach to a large collection of proteins from sequenced genomes. First, we initialized our database by downloading the December 2009 snapshot of annotated genomes available in the JGI IMG database. This data set includes 1,741 Bacterial genomes, 77 Archaeal genomes and 76 Eukaryotic genomes. Of the 1,894 genomes in the first iteration of the database, 992 are finished genomes, 898 are draft assemblies and 4 are permanent drafts. There are 7,165,275 annotated protein sequences associated with these genomes, which served as the input to our clustering workflow.

Generating *de novo* protein families requires calculation of the similarity of each pair of proteins, a procedure that grows as a function of the square of the sequence database size. Performing this calculation on over 7 million proteins requires significant time and memory. To reduce the computational complexity of this pairwise sequence-similarity search, we applied our iterative approach to sift through genomes and define protein families. We start with large, well-defined families and sequentially add in the remaining proteins. Because the vast majority of the available sequence data is from Bacteria and Archaea (88.5% of total protein sequences) and because genomes from these groups have been selected for sequencing in a phylogenetically informative manner [5], we limited our initial cluster analysis to 313,139 proteins from 100 phylogenetically diverse representative Bacterial and Archaeal genomes (Additional file 1). We clustered these proteins using MCL and identified 23,336 protein families, 720 of which were present in at least 50% of the 100 representative genomes. These widely distributed families tend to be large, well studied, phylogenetically diverse, and well conserved across the Bacteria and Archaea (Additional file 2). We built profile HMMs of these families and used them with the hmmsearch algorithm to identify additional family members in the full set of ~7 million proteins. We found that the 720 widely distributed families recruited 1,541,029 sequences.

**Figure 2:**
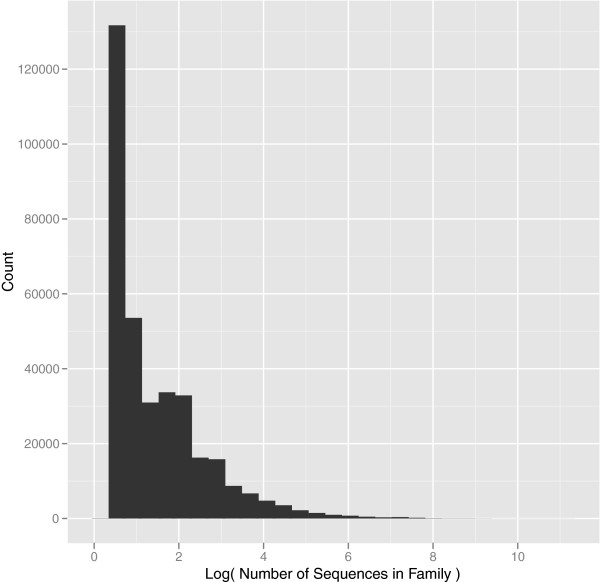
**SFam Size Distribution. **The distribution of SFam size, measured as the number of sequences belonging to each SFam, is illustrated in this histogram. The x-axis represents the log SFam size while the y-axis represents the number of SFams of a given size.

Next, we subjected the remaining 5,624,246 proteins to a pairwise similarity search, which took approximately 76,000 cpu hours. We found that 4,557,723 sequences exhibit significant similarity to at least one other sequence in the database. This similarity data was used as input into MCL to cluster sequences into SFams. Prior to running MCL, we filtered this pairwise similarity data using alignment coverage to mitigate sequence length-dependent clustering biases and to ensure that all members of an SFam are global homologs. Specifically, the pairwise alignment had to include at least 80% of both sequences or their similarity was set to 0. Applying MCL to this coverage-filtered data grouped the sequences into 344,921 SFams. SFams ranged in size from 2 to 45,609 members and are composed of global homologs. Most SFams were small, suggesting that they are phylogenetically restricted or not well represented by the current sampling of genomes (Figure 2). The biological annotations that are enriched among the largest families reinforce the hypothesis of phylogenetic restriction; most appear to be relatively well-studied and involved in general cellular maintenance (e.g. ABC transporter (IPR010067), DNA Polymerase III (IPR006054), etc.) and are thus likely common to most genomes (p<0.05; Additional file 3). However, some of the annotations that are enriched among large families appear to be more specific in their activity and are thus less likely to be found in most genomes (e.g., Drug resistance transporter EmrB/QacA (IPR004638), Disease resistance protein (IPR000767), etc.). SFams with such annotations may have been subject to relatively large copy number expansions. Future work will thoroughly explore the evolution of SFam size across the tree of life.

We then generated multiple sequence alignments, phylogenetic trees and HMMs for each SFam. Because large multiple sequence alignments can be prone to error [22], we developed a method that selects diverse representative sequences from a family (Methods). Representatives were used to generate a training alignment for those SFams with more than 250 members (N=3,084). All other family sequences were aligned to this training alignment before building HMMs and phylogenies for these large families.

Our iterative MCL clustering approach assigned 85.1% of the proteins subject to clustering to an SFam. The remaining 1,066,523 sequences are either ORFans, misannotated sequences (e.g., sequence truncations due to gene splitting), or too diverged from the other family members for homology to be inferred via sequence comparison. Notably, many of these sequences have significant local alignments to other sequences, suggesting that they may share common and promiscuous protein domains.

### Requiring high coverage identifies truncated and novel sequences

Because we designed SFams to represent globally homologous proteins, the step in our workflow that calculates the similarity between every pair of sequences only does so if their alignment covers at least 80% of both sequences. As a result of this threshold, 801,279 sequences were removed from the clustering analysis, many of which exhibited significant local pairwise alignments with other sequences. We characterized these filtered sequences by comparing them to each family’s HMM and analyzing the alignments.

We found that 27.4% of the filtered sequences (N=219,707) display evidence of being relatively truncated (e.g., fragmented) members of at least one family. Specifically, these sequences have significant alignments with a relatively long HMM (i.e., one that is at least 20% longer) and the alignment includes at least 80% of the filtered sequence. Conversely, 16.9% (N=135,841) of the sequences are longer than any of the HMMs they significantly align with, suggesting that these sequences may have been subject to an evolutionary event (e.g., gene fusion, expansion of repetitive elements) or annotation error that increased the sequence’s length. The remaining 55.7% of the filtered sequences may represent novel protein families for which only a single member has been identified, or highly diverged members of other protein families.

### SFam HMMs can be used to accurately identify new family members

We evaluated the ability of each family’s HMM to recruit new family members by comparing the initial 7,165,275 input protein sequences to each of the 345,641 HMMs. Sequences were recruited into families if they could be globally aligned to a SFam’s HMM. We then used recall and precision metrics to estimate how well an HMM exclusively detects all members of the family it represents (i.e., family members). For example, a recall equal to 1.0 indicates that the HMM detected all of its family members, suggesting that the HMM accurately recruited members of the family and that the family members are generally global homologs of one another. A precision equal to 1.0 indicates that the HMM only recovered its own family sequences, suggesting that the HMM differentiated family members from non-family members and that the family members are generally not homologs of members of other families.

**Figure 3:**
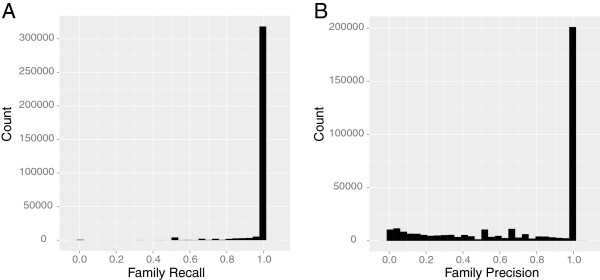
**SFam Precision and Recall. **The SFam **(A)** recall and **(B)** precision distributions are illustrated in these histograms. The x-axis represents recall or precision values while the y-axis represents the number of SFams of a given value.

Figure 3 illustrates the distribution of precision and recall across SFams. The large mass of SFams with a recall near 1.0 indicates that the families generally include only sequences that are detectably homologous to one another across their entire length using HMM profile searches. Indeed, roughly 90% of the SFams have a recall of 1.0 (N=312,966) and the average SFam has a recall of 0.99. However, this estimate of recall does not account for novel sequences, because the test sequences that we classify into SFams were all used during HMM construction. To estimate recall for novel sequences, we conducted a leave-one-out analysis, wherein the sequence being tested is removed from its family’s alignment and the HMM for that SFam is recomputed. Since it is computationally intractable to perform a leave-one-out analysis every sequence from every family, we analyzed 3,500 randomly sampled families that contain at least three sequences to leave-one-out recall tests (N~50,000 sequences). We find that SFam leave-one-out recall is high (mean=0.97) and exhibits a significant positive correlation with its standard recall (Pearson’s r = 0.59; p<10^-15^). These results suggest that standard recall serves as a good proxy for the leave-one-out recall and that SFam HMMs are generally capable of identifying homologs of the family.

The distribution of SFam precision values has a larger spread, indicating that a subset of SFams are derived from sequences that are not highly differentiated from each other. While approximately 58% of the families have a precision of 1.0 (N=199,913), there is a long tail of lower-precision families. Indeed, roughly 35% of the families (N= 121,409) have a precision less than 0.75. These families are enriched for several biological functions that are well distributed across the tree of life, frequently found in high copy number per genome, and often curated into superfamilies (i.e., large clusters composed of distinct sub-clusters) in various protein family databases (p<0.05; Additional file 4). These include various transport proteins (e.g., ABC Transporter (IPR010067), Permease (IPR005495), Secretin/TonB (IPR011662), etc.) and proteins involved in cytochrome assembly (e.g., cytC (IPR012127), Cytochrome b/b6 (IPR005797), Cytochrome P450 (IPR001128), etc.). These results suggest that a subset of SFams share similarity with other SFams and that they may be over partitioned. As evidenced by the functional enrichment data, many of the low-precision SFams may actually represent subfamilies (i.e., sub-clusters) within a superfamily. Over 54% of the families have a precision and recall of 1.0, suggesting that, in general, SFams are well partitioned and can be used to accurately identify their members. We include the results of this precision and recall analysis for each family as a qualifier in our database.

### SFams are related through a sparse network

The precision results indicate that some SFams recruit non-member sequences. This may result from an over-partitioning of the data, wherein some SFams share an evolutionary history with other families. For example, two SFams may be distinct sub-clusters (i.e., subfamilies) within a larger family (i.e., superfamily). We emitted an HMM consensus sequence for each SFam and aligned all pairs of consensus sequences to evaluate the relationship between SFams. We found that 33% of our SFams do not exhibit significant sequence similarity with any other family, suggesting that they are well partitioned and distinct. The remaining 67% of SFams have similarity to at least one other family, indicating that some of the SFams are either over-partitioned (at an MCL inflation value of 2) or not phylogenetically distinct (e.g., distinct families that share common protein domains).

**Figure 4:**
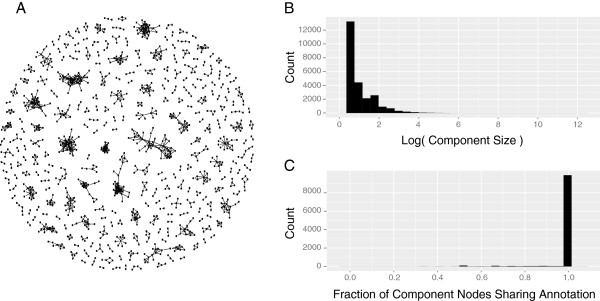
**SFams are Related Through a Sparse Network. **Comparing the similarity between SFams reveals a network of relationships between families. The components that comprise the network tend to have a minimally connected topology, as illustrated by a random sampling of 250 components from the network **(A)**. Here, SFams are represented by dots and lines represent relationships between SFams. Most components are populated by a small number of SFams, as illustrated in the component size distribution (**B**; log scale). Most components are made up of SFams that share at least one functional annotation, as indicated in the distribution of the fraction of nodes per component that share an annotation label **(C)**.

We evaluated the structure of these relationships through network analysis. We represented families as nodes (N=231,107) and relationships of significant similarity between families as unweighted and undirected edges (N=1,177,242). We evaluated the network topology by calculating various network summary statistics, including the median node (Additional file 5) degree (4.0), betweenness (3.0), transitivity (0.24), and closeness (0.12). The distributions of the degree and betweenness summary statistics are characterized by a long tail, which indicates that most SFams share similarity with a small number of families. We also found that the network contains 24,674 distinct components (i.e., sub-networks), many of which may represent superfamilies. Most are composed of two SFams (Figure 4), though one component is especially large, containing 54% of the nodes. The network summary statistic distributions for this large component are similar to those of the entire network, though the median node is slightly more connected (median degree = 7) and the average node is skewed towards a higher connectivity (Additional file 6). This suggests that while the topology of this large component is analogous to the entire network, it contains an enrichment of highly connected nodes compared to the other components.

This analysis reveals that most SFams have few direct relationships to other SFams and represent peripheral nodes within the network. A relatively small number of SFams represent network hubs. High-degree SFams are enriched for several functions (p<0.05; Additional file 7), including signal transduction [e.g., Signal transduction histidine kinase-related protein (IPR004358), response regulator (IPR001789), and core (IPR005467); PAS (IPR000014), etc.], membrane transport [e.g., ABC transporter-like (IPR003439); Type II secretion system gspD (IPR004845); and ABC Transporter, permease protein (IPR000522)], and sugar metabolism [e.g., Carbohydrate kinase (IPR000577), various Glycoside hydrolases (IPR008270, IPR000322)]. Some functions that are enriched among high-degree SFams are also enriched among low-precision SFams, especially those functions associated with ribosome biogenesis [e.g., Ribosomal proteins S5 (IPR000851), S18 (IPR018275) and L30 (IPR018038)]. In total, these network results indicate that while the majority of our SFams share sequence similarity with at least one other SFam, most are not similar to more than two other SFams. Hence, a user wanting to annotate a novel sequence via comparison to our SFam HMMs may want to evaluate two or three of the top hits, but generally does not need to consider a large number of hits.

We use the results of this consensus-sequence network analysis to partition SFams into sets of families that are related through sequence similarity (*i.e.,* clans). Clans can be used to improve the interpretation of SFam search results. For example, when using SFams to functionally annotate a new sequence, one might place greater weight on the putative annotation for a sequence that is recruited into multiple SFams within the same clan relative to a sequence that is recruited into multiple SFams that are distributed across multiple clans. To provide flexibility to users of our database, we also provide a listing of SFam clans that is based on HMM recruitment of member sequences. Specifically, if two families reciprocally recruit 80% of one another’s member sequences, then we merge these families into a clan using single-linkage clustering. This procedure finds that 236,542 families are recruited into one of the 26,253 clans that this analysis identifies. Both sets of clans are made available to users of our database.

### The majority of SFams are associated with Interpro annotations

We functionally annotated each SFam by assigning Interpro [23] annotations to the families on a majority-rules basis. We find that 52% of the families (N=180,646) have at least one Interpro annotation (Additional file 8). An additional 43,395 SFams (12%) contain at least one Interpro-annotated sequence, but not enough to meet the majority-rules threshold. The remaining families have no described functional annotation, suggesting that their function is unknown. We also evaluated the functional consistency of each network component by calculating the fraction of annotated component SFams that share an Interpro annotation. We found that the majority of components have at least one Interpro annotation identifier that is common to all annotated SFams within the component (Figure 4).

These functional annotations can be used to rapidly annotate new genomes with fairly high confidence by classifying the protein sequences into SFams and transferring the annotation. However, similarity-based annotation can yield erroneous inferences of function when similarity and function are not tightly linked [24]. Thus, we recommend using only the 39% of the SFams (N=135,929) that contain sequences which all share the same functional Interpro identifier when inferring the function of a new sequence.

### SFams cover most diversity in new genomes and can be automatically extended to incorporate new families

To illustrate how our SFam database can be used to annotate new protein sequences, we executed a second iteration of our workflow to classify protein sequences obtained from recently released microbial genomes into SFams. Specifically, we evaluated 3,394,628 predicted proteins obtained from 1,079 genomes that were submitted to the JGI IMG database between December 2009 and August 2011. Each protein was compared to each of the 345,641 SFam HMMs identified in the first iteration of our workflow. Roughly 84% of the sequences (N=2,866,297) were assigned to an SFam currently defined in our database, and 73% (N=2,101,401) were recruited into a functionally annotated family. This high rate of annotation demonstrates the utility of our SFam identification procedure and suggests that our SFams cover a large percentage of protein diversity in recently sequenced genomes.

Nonetheless, a substantial number of protein families remain to be identified. Our automated, iterative MCL procedure provides a means for defining new SFams from the unannotated sequences. After sifting out all sequences that show homology to one of the existing SFams, we subjected the remaining 528,331 proteins from recently sequenced genomes plus the 801,279 proteins that were not clustered into an SFam in our initial analysis to an independent round of pairwise BLASTP and MCL, as described above. This *de novo* clustering identified an additional 90,719 SFams. These SFams and their member sequences were added to our database and can be differentiated from the previously identified SFams through a unique identifier (*familyconstruction_id* = 3). As additional genomes are sequenced, this procedure will be repeated to expand the diversity of SFams encoded in our database. This analysis demonstrates how our SFam database can be rapidly and automatically updated.

### SFams can be used to rapidly and thoroughly annotate metagenomic sequences

Our database of SFams can also be used to annotate metagenomes. For example, metagenomic sequences can be compared the database of HMMs to identify homologs of SFams. In their study of the *Sorcerer II* Global Ocean Sampling (GOS) metagenome, Yoosef et al. [6] predicted proteins from assembled metagenomic reads using two methods: (1) *de novo* clustering of translated open reading frames predicted from metagenomic sequences, along with full-length reference proteins and (2) annotation of translated metagenomic ORFs using HMMs from Pfam [12] and TIGRFAM [13]. The authors discovered that while *de novo* clustering was computationally expensive (>1 Million CPU hours), it resulted in substantially more predicted proteins than did the HMM based strategy.

We evaluated the performance of SFams on the annotation of metagenomic data by classifying predicted GOS metagenomic peptides into SFams. Specifically, we downloaded from CAMERA [25] 6,115,812 peptides that were predicted in the GOS study [26] and compared each peptide to each of the 345,641 first-iteration SFam HMMs in our database using hmmscan. We classified 80.4% of the sequences (N=4,919,769) into one of our SFams. Of these sequences, 92.3% (N=4,540,966) were classified into a functionally annotated SFam. These percentages exceed the 63% of proteins that we were able to annotate when using Pfam (v 26.0) and TIGRFAM (v 12.0) HMMs, indicating that our SFams cover a greater diversity of marine metagenome proteins than other protein family databases. Importantly, we can fairly thoroughly annotate a metagenome with substantially fewer computational requirements than *de novo* clustering. As metagenomic studies grow in size due to advances in technology and dropping sequencing costs, the classification of metagenomic sequences into reference SFams will be more tractable than performing *de novo* protein family clustering for every study.

### Resources and availability

Our SFam database and software workflow is publicly available for download (http://edhar.genomecenter.ucdavis.edu/sifting_families/). This database includes the ~10.5 million protein sequences obtained from the 2,928 genomes that we analyzed across the two iterations of our workflow. It also includes multiple sequence alignments, HMMs and phylogenetic trees describing each of the 436,360 SFams that we characterized. We also make available various details on the SFams, including precision, recall, clan listings and functional annotation. As additional genome sequences become available, we will expand the diversity of the SFams described in our database through iteration of the workflow. Our procedure ensures that our knowledge of known protein family diversity will grow at a rate consistent with the rate at which new genomes are discovered.

We recognize that some of the large-scale computational analyses that we conducted produced data that may be useful for other researchers. Thus, in addition to the SFam database, we provide access to several large datasets, including the results of the initial pairwise sequence–similarity search (~7 million proteins), the pairwise similarity matrix used as input into MCL, the MCL result files (Inflation = 2), and the SFam similarity network. Researchers can use these data sets to explore the relationship between proteins or SFams or build their own families using MCL with alternative inflation values or different *de novo* clustering algorithms.

## Conclusions

We developed an automated, high-throughput procedure that iteratively identifies SFams from whole genome sequences. We used this procedure to populate a database of SFams that provides a basis for describing the diversity of protein families and functions. Our approach streamlines the identification of novel protein families, ensuring that the diversity described in our database of SFams will keep pace with the diversity discovered through additional genome sequencing. The approximately 30% of SFams that do not have an Interpro identifier suggests that our database contains novel diversity relative to other commonly used HMM-based protein family databases. SFams should thus prove to be a useful supplemental resource for analyses that rely on classifying sequences into families. Indeed, we found that our procedure facilitates rapid and thorough annotation of genome and metagenome sequences.

Although this approach provides an updateable and comprehensive dataset of families, our sequence similarity–based SFams are limited because they are not built based on functional relatedness. Additionally, SFams are identified using high-throughput procedures, which may trade sensitivity for accuracy. For example, many SFams, especially those with low precision or a large number of degrees, may represent subfamilies within a superfamily. Given that we apply a single MCL inflation value to all SFams that we identify, some over-partitioning of the data during *de novo* clustering is expected. In addition, our selection of various thresholds that are used to infer homology between a pair of sequences or a sequence and a family may produce family-composition errors. Fortunately, our statistical assessments indicate that most SFams are well partitioned and only exhibit similarity with no more than one other SFam. Furthermore, we use previously identified families to reduce the computational complexity of identifying novel SFams. This could introduce errors, such as in the case where two proteins from the same family are recruited into two separate previously identified families. Our decision to maximize the phylogenetic diversity of the set of genomes that we used to initialize our database of families should mitigate – though it cannot eliminate – this potential problem. Regardless, as a result of these aforementioned properties of our SFam database, downstream curation is required for SFams of interest. We provide access to our pairwise similarity matrix as well as our SFam statistics and network relationships to aid users of our database in the curation of specific SFams.

We plan to provide regular updates to the SFam database as new genome sequences become available. In addition, as the phylogenetic representation of Eukaryotic genome sequences improves, we will identify those SFams that are widely distributed across Eukarya. Future work will also include automatically detecting superfamily and subfamily SFams. Additionally, we plan to characterize the phylogenetic and ecological distributions of SFams. Our ultimate hope is that through adoption of high-throughput and computationally efficient procedures such as the identification of SFams, researchers can leverage the inundation of biological sequence data to thoroughly and rapidly characterize the diversity of proteins in nature.

## Methods

### Identification of widely distributed protein families

We use representative genomes and widely distributed proteins to seed the iterative clustering of protein families. First, we selected 100 representative bacterial and archaeal genomes in a phylogenetically informative manner (SI Table 1). To identify archaeal representatives, we retrieved the translated RadA gene sequence from 49 completed archaeal genomes, aligned these sequences using MUSCLE (v3.8) [27], and constructed a phylogenetic tree using PHYML with the JTT model [28]. This tree was used to sort genomes by their phylogenetic distance (PD) contribution [5]. The top 15 archaeal PD contributors were selected to be the representatives for the domain. Similarly, to identify bacterial representatives, we used a phylogenetic tree constructed from a concatenated alignment of 31 bacterial marker genes [5]. The bacterial genomes were sorted by their PD contribution and the top 85 genomes were selected as representatives.

The 313,139 proteins encoded in these 100 genomes were used to define an initial collection of protein families. We calculated the similarity between all pairs of proteins using BLASTP (v2.2.22) [26]. We applied two thresholds to the BLASTP results to infer whether a pair of sequences is homologous across their entire length: the pairwise alignment must produce an e-value no greater than 10^-10^ and cover 80% of the lengths of both proteins. We apply particularly stringent e-value threshold at this step (compared to the steps below) to ensure that the families that we identify as being widely distributed across the Bacteria and Archaea are designated as such due to a high degree of sequence similarity and not a relaxed cutoff. Alignments that did not meet these requirements were discarded and the similarity between the pair of sequences was set to zero.

We used MCL (v1.008) [21] with an inflation value of 2 to *de novo* cluster proteins into homologous families based on the similarity between all pairs of proteins. This procedure identified 23,336 families with two or more members. We then calculated the universality score for each family, which is the percentage of the representative genomes that have at least one member of the family of interest. Widely distributed protein families were defined as those families that span at least 50% of the 100 representatives. We identified a total of 720 widely distributed families.

Next, we constructed an alignment and a profile-Hidden Markov model (HMM) for each widely distributed family. Because the families tend to be composed of many proteins (N > 250), we performed these steps with a diverse subset of sequences for each family. For each family, we first clustered member sequences into *de novo* subfamilies by subjecting the family’s BLASTP similarity data to MCL. Here, we included as input to MCL only those pairs of sequences that were no greater than 80% similar. If this procedure divided a family into more than 200 subfamilies, we iteratively decreased the similarity threshold and repeated the process until fewer than 200 subfamilies were identified. One member sequence was randomly selected from each subfamily to serve as a representative sequence. Representative sequences were aligned using MUSCLE. Alignment columns containing more than 80% gaps were excised from the alignment. HMMs were built from the trimmed alignments using hmmbuild (HMMER v3.0, March 2010) [29]. These alignments were also used to build phylogenetic trees using FastTree [30].

### Using widely distributed gene family HMMs to reduce sequence database size

We identified and filtered (i.e., “sifted”) homologs of widely distributed families to reduce the size of the sequence database subject to *de novo* clustering. We downloaded the December 2009 snapshot of the JGI’s Integrated Microbial Genomes database [31], which contains 7,168,765 protein sequences distributed across 1,894 annotated genomes. We then used hmmscan (HMMER3) to compare these sequences to the 720 widely distributed family HMMs [29]. Proteins that matched an HMM with an e-value less than 10^-5^ and that produced a match that includes at least 80% of the residues from both the sequence and the HMM were designated as members of that family and were excluded from the subsequent clustering analyses. We use the HMM alignment envelope coordinates to calculate the number of HMM positions that match the sequence. These widely distributed families and their member sequences, including those that were identified in this analysis of 1,894 genomes, are included in our database in the *Families* and *Familymembers* tables, respectively. They are differentiated from the other families we constructed via a unique identifier (*familyconstruction_id* = 1).

### Identification of SFams via *de novo* clustering

All remaining unfiltered sequences were compared to one another using BLASTP. We inferred that a pair of proteins are homologous across their entire length if their BLASTP alignment covered at least 80% of both sequences and produced an e-value no greater than 10^-5^. For these homologous pairs of sequences, we defined similarity as their percent sequence identity. The similarity for remaining pairs was set to zero. We used this relatively sparse similarity matrix to cluster sequences into SFams via MCL (Inflation = 2). Clusters containing a single sequence were not considered SFams.

Multiple sequence alignments, phylogenetic trees and HMMs were generated for each SFam. For SFams larger than 250 sequences, we identified representative sequences using the method described above. For each family, sequences were aligned using MUSCLE [27]. Phylogenetic trees and HMMs were generated from the multiple sequence alignments via FastTree [30] and hmmbuild [29], respectively. These *de novo* families and their member sequences are available through our database in the *Families* and *Familymembers* tables, respectively. They are identified through a unique index (*familyconstruction_id* = 2).

### Analysis of protein family quality and similarity

We evaluated the quality of each SFam’s HMM by using hmmscan (HMMER3) to compare each HMM to each protein sequence in our database. Sequences were recruited into families if their alignment to a SFam’s HMM had an e-value no greater than 10^-5^ and covered at least 80% of both the sequence and the HMM. Recruited sequences were used to calculate the precision and recall of each SFam’s HMM, where the precision is the fraction of total sequences recruited by the HMM that are family members, and the recall is the fraction of family members that are recruited by the HMM.

We compared the similarity between SFams by comparing HMM consensus sequences. We used hmmemit (HMMER3) [29] to generate each family’s consensus sequence. All pairs of consensus sequences were compared using BLASTP. Homology was inferred between a pair of consensus sequences if their alignment had an e-value no greater than 10^-5^ and covered at least 80% of the shorter sequence in the pair. We used the R package igraph [32] to analyze the network of homologous relationships between SFams.

### Identification of fragmented family members

Not all sequences that were obtained from the initial genomes were clustered into an SFam. These sequences may represent novel SFams. Alternatively, many may be fragmented or incompletely sequenced members of families that were separated from the family through the filtering criteria of our bioinformatics pipeline. To differentiate fragmented SFam members from representatives of putatively novel SFams, we used hmmsearch (HMMER 3.0) to compare each non-clustered sequence to each SFam’s HMM. Sequences were considered SFam member candidates if they aligned to a consensus sequence with a maximum e-value of 10^-5^ and if the alignment covered at least 80% of either sequence. We do not include family member candidates in our database of SFams.

### Functional annotation of SFams and identification of enriched annotations

The member sequences for each SFam were functionally annotated using Interpro. We obtained Interproscan [23] annotations for each sequence from the JGI’s IMG Database. Sequence-level annotations were mapped onto family-level annotations if a majority of sequences in the SFam support the annotation.

We conducted tests of independence to identify functions that are statistically associated with various family properties (e.g., family size, precision, and degrees). The distributions of family properties were used to partition the families for each property. Large families and high-degree families were designated as those in the fourth quartile of their respective distributions. Low precision families were designated as those with a precision less than 0.75. We then constructed two-by-two contingency tables for each Interpro annotation by counting the number of families that do and do not possess the annotation as well as the number of families that are and are not in the property partition of interest. We used these tables to conduct one-sided tests of independence in R using the fisher.test function. We applied a Bonferroni correction to account for multiple tests.

## Competing interests

The authors declare that they have no competing interests.

## Authors’ contributions

TJS designed the database, conducted analyses of SFam precision, recall, and network topology, annotated the GOS metagenome with SFams, and conceived of and designed experiments. GJ designed the database, populated the database, identified the similarity between all pairs of proteins, identified fragmented proteins and assembled a network of SFams. DW identified the widely distributed families, annotated new genomes using SFams and designed experiments. MGIL designed the database, populated the database, and conceived of and designed experiments. JAE and KSP designed the database and conceived of and designed experiments. All authors contributed to the writing of the manuscript. All authors read and approved the final manuscript.

## Funding

Funding for this work was provided by the Gordon and Betty Moore Foundation (grant #1660 and #3300 to KSP and JAE, http://www.moore.org/), NSF (grant #DMS-1069303 to KSP), and a gift to the Pollard lab from the San Simeon Fund.

## Supplementary Material

Additional file 1 Representative genomes used in this study. Click here for file

Additional file 2 Widely distributed protein family statistics. Click here for file

Additional file 3 Interpro annotations enriched among the quartile of SFams with the largest size. Click here for file

Additional file 4 Interpro annotations enriched among SFams with a precision less than 0.75. Click here for file

Additional file 5 **Distributions of various network topology statistics for the entire SFam similarity network.** Each histogram illustrates the distribution of a network statistic for each node in the SFam similarity network, including degree centrality (upper left, log scale), betweenness centrality (upper right; x-axis scale constrained at a betweenness of 50), transitivity (lower left), and closeness centrality (lower right). Click here for file

Additional file 6 **Distributions of various network topology statistics for the largest SFam similarity component.** Each histogram illustrates the distribution of a network statistic for each node in the largest SFam similarity network component, including degree centrality (upper left, log scale), betweenness centrality (upper right; x-axis scale constrained at a betweenness of 50), transitivity (lower left), and closeness centrality (lower right). Click here for file

Additional file 7 Interpro annotations enriched among the quartile of SFams with the highest number of degrees. Click here for file

Additional file 8 The distribution of the number of Interpro annotations detected per SFam. Click here for file
