# Supplementary figures and images for: Sifting through genomes with iterative-sequence clustering produces a large, phylogenetically diverse protein-family resource
Source: BMC Bioinformatics. 2012 Oct 13;13:264. doi: 10.1186/1471-2105-13-264 (PMC3481395; doi:10.1186/1471-2105-13-264)

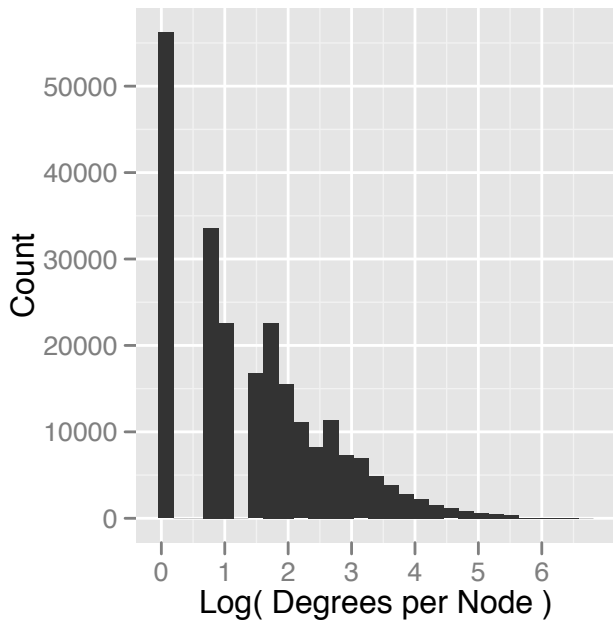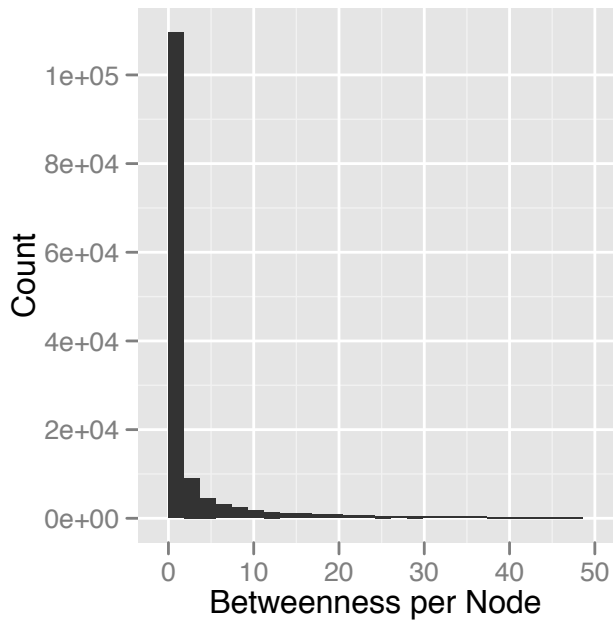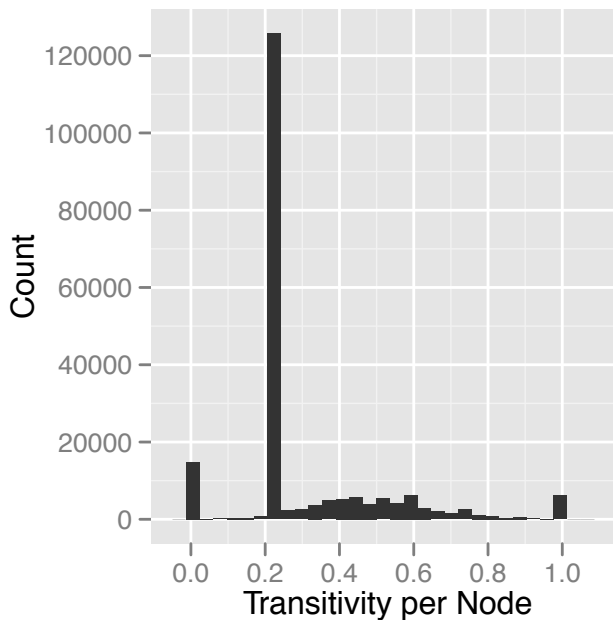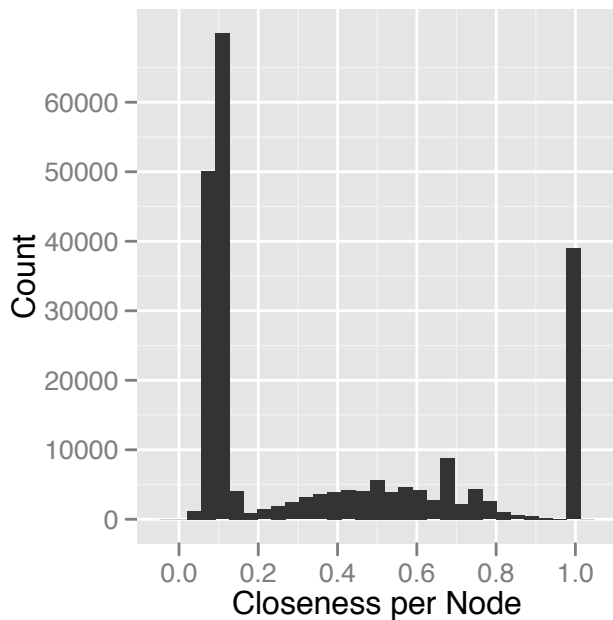

Supplement: Additional file 5 — Distributions of various network topology statistics for the entire SFam similarity network. Each histogram illustrates the distribution of a network statistic for each node in the SFam similarity network, including degree centrality (upper left, log scale), betweenness centrality (upper right; x-axis scale constrained at a betweenness of 50), transitivity (lower left), and closeness centrality (lower right). [file 1471-2105-13-264-S5.pdf]

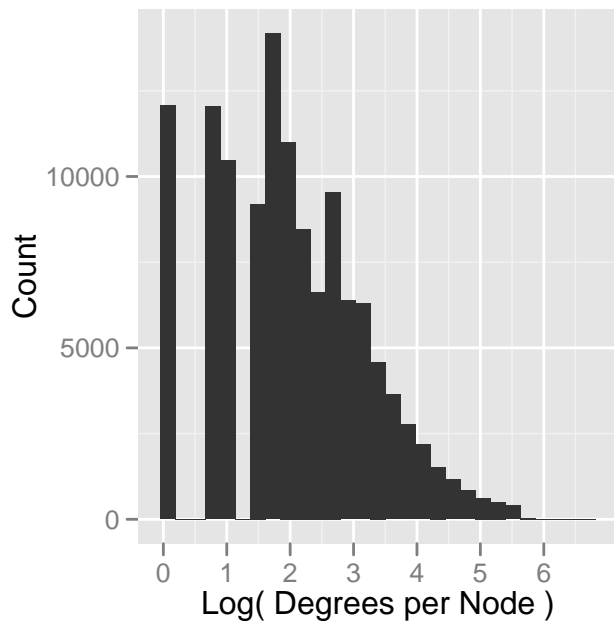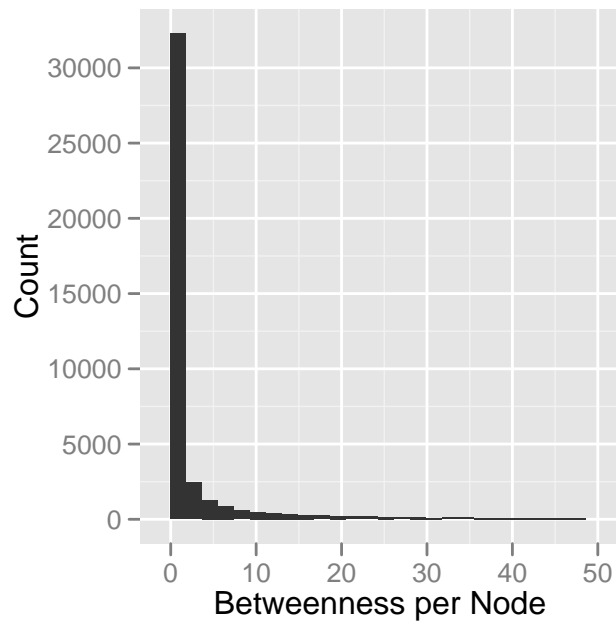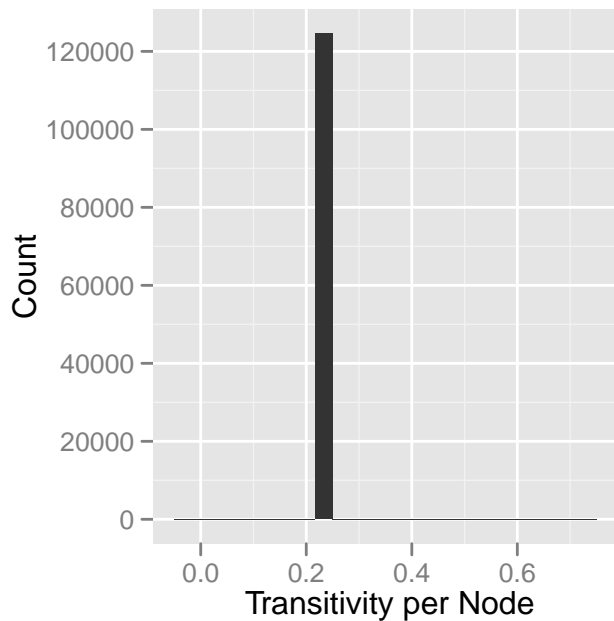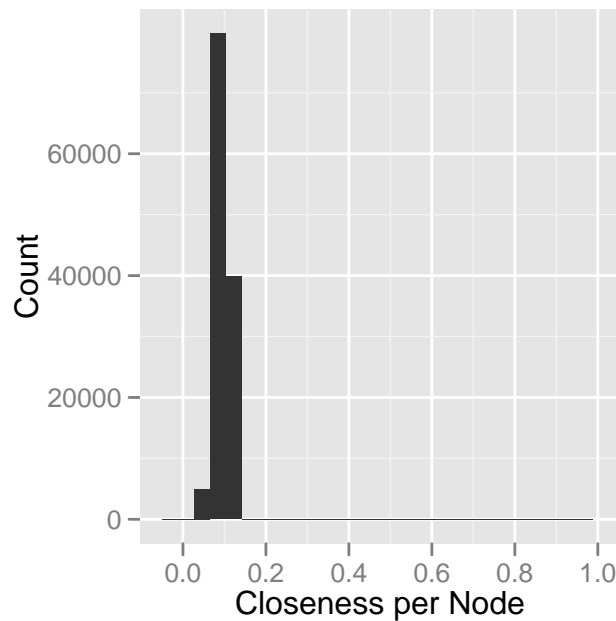

Supplement: Additional file 6 — Distributions of various network topology statistics for the largest SFam similarity component. Each histogram illustrates the distribution of a network statistic for each node in the largest SFam similarity network component, including degree centrality (upper left, log scale), betweenness centrality (upper right; x-axis scale constrained at a betweenness of 50), transitivity (lower left), and closeness centrality (lower right). [file 1471-2105-13-264-S6.pdf]

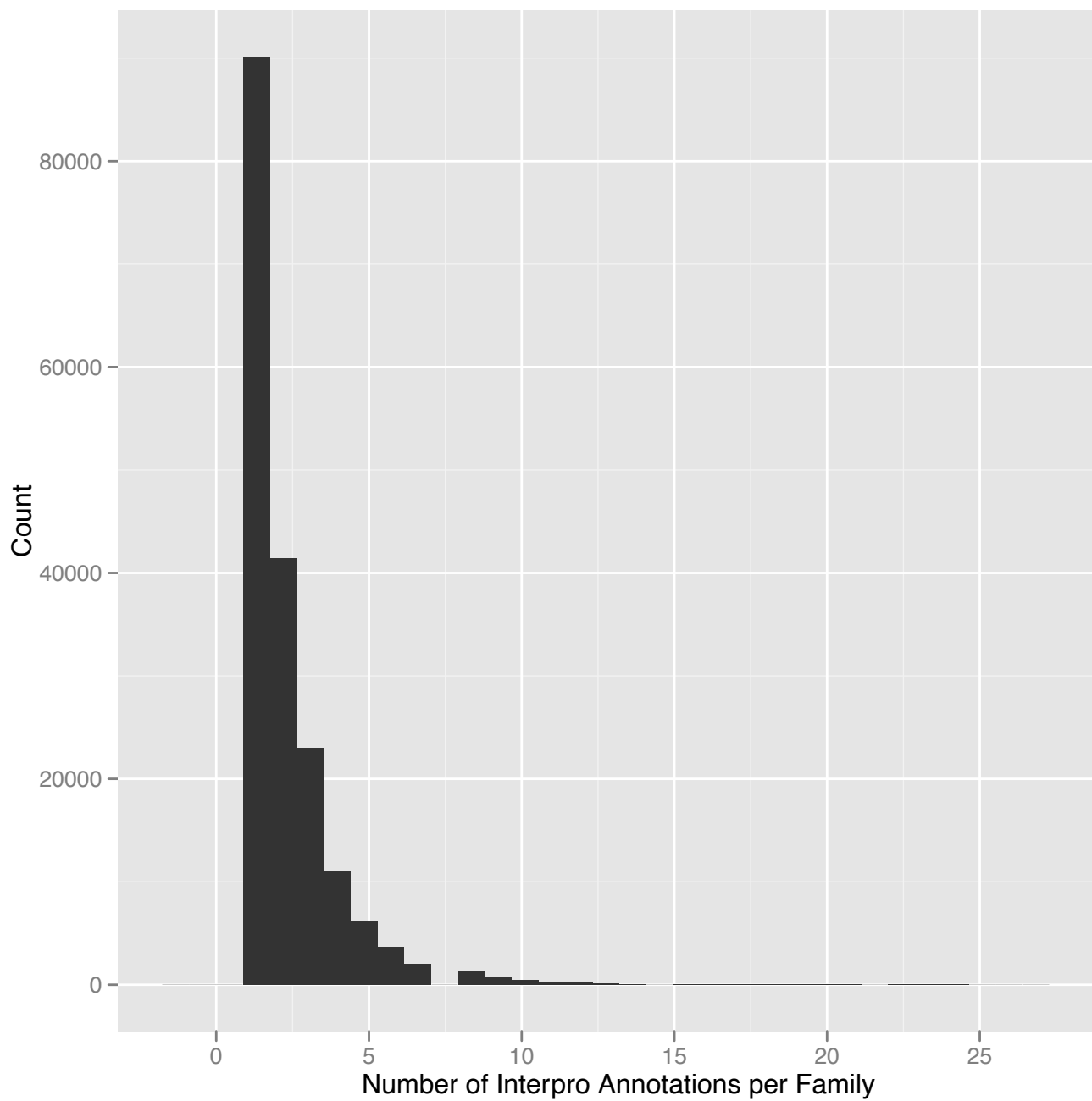

Supplement: Additional file 8 — The distribution of the number of Interpro annotations detected per SFam. [file 1471-2105-13-264-S8.pdf]
